# Endothelial colony‐forming cell therapy for heart morphological changes after neonatal high oxygen exposure in rats, a model of complications of prematurity

**DOI:** 10.14814/phy2.13922

**Published:** 2018-11-28

**Authors:** Camille Girard‐Bock, Carla C. de Araújo, Mariane Bertagnolli, Thuy‐An Mai‐Vo, Arul Vadivel, Rajesh S. Alphonse, Shumei Zhong, Anik Cloutier, Megan R. Sutherland, Bernard Thébaud, Anne Monique Nuyt

**Affiliations:** ^1^ Department of Pediatrics Sainte‐Justine University Hospital Research Center Faculty of Medicine Université de Montréal Montreal Quebec Canada; ^2^ Ottawa Hospital Research Institute University of Ottawa Ottawa Ontario Canada; ^3^ Department of Pediatrics University of Alberta Edmonton Alberta Canada; ^4^ Present address: Centre Intégré Universitaire de Santé et de Services Sociaux du Nord‐de‐l’Île‐de‐Montréal Hôpital du Sacré‐Cœur de Montréal Research Center Université de Montréal Montréal Quebec Canada; ^5^ Present address: Monash Biomedicine Discovery Institute Department of Anatomy and Developmental Biology Monash University Clayton Victoria Australia

**Keywords:** Cell therapy, heart, oxygen‐induced cardiomyopathy, preterm birth

## Abstract

Very preterm birth is associated with increased cardiovascular diseases and changes in myocardial structure. The current study aimed to investigate the impact of endothelial colony‐forming cell (ECFC) treatment on heart morphological changes in the experimental model of neonatal high oxygen (O_2_)‐induced cardiomyopathy, mimicking prematurity‐related conditions. Sprague–Dawley rat pups exposed to 95% O_2_ or room air (RA) from day 4 (P4) to day 14 (P14) were randomized to receive (jugular vein) exogenous human cord blood ECFC or vehicle at P14 (*n* = 5 RA‐vehicle, *n* = 8 RA‐ECFC,* n* = 8 O_2_‐vehicle and *n* = 7 O_2_‐ECFC) and the hearts collected at P28. Body and heart weights and heart to body weight ratio did not differ between groups. ECFC treatment prevented the increase in cardiomyocyte surface area in both the left (LV) and right (RV) ventricles of the O_2_ group (O_2_‐ECFC vs. O_2_‐vehicle LV: 121 ± 13 vs. 179 ± 21 *μ*m^2^, RV: 118 ± 12 vs. 169 ± 21 *μ*m^2^). In O_2_ rats, ECFC treatment was also associated with a significant reduction in interstitial fibrosis in both ventricles (O_2_‐ECFC vs. O_2_‐vehicle LV: 1.07 ± 0.47 vs. 1.68 ± 0.41% of surface area, RV: 1.01 ± 0.74 vs. 1.77 ± 0.67%) and in perivascular fibrosis in the LV (2.29 ± 0.47 vs. 3.85 ± 1.23%) but in not the RV (1.95 ± 0.95 vs. 2.74 ± 1.14), and with increased expression of angiogenesis marker CD31. ECFC treatment had no effect on cardiomyocyte surface area or on tissue fibrosis of RA rats. Human cord blood ECFC treatment prevented cardiomyocyte hypertrophy and myocardial and perivascular fibrosis observed after neonatal high O_2_ exposure. ECFC could constitute a new regenerative therapy against cardiac sequelae caused by deleterious conditions of prematurity.

## Introduction

Worldwide, about 11% of infants are born preterm (Liu et al. 2016). While the rate of preterm birth has remained stable in the last few years, advances in perinatal and neonatal care over the past decades have markedly improved survival of premature infants so that the absolute numbers of adolescents and young adults born preterm are increasing (Blencowe et al. 2013; Borghesi et al. 2013; Martin et al. 2017).

Premature infants are exposed to a wide range of intrauterine and neonatal insults, such as oxidative stress, ischemia or sepsis, striking at critical periods of organ development (Nuyt et al. 2017). Subsequent morphological and functional fragilities may influence long‐term health and translate into adult illness including hypertension and cardiovascular disease (Boivin et al. 2012; Luu et al. 2016, 2017; Nuyt et al. 2017; Raju et al. 2017). Cardiac shape alterations and systolic and diastolic function impairments have been reported in young adults born preterm (Lewandowski et al. 2013a,b; Kowalski et al. 2016, 2018). Mimicking the findings from clinical studies, experimental models of preterm birth‐related conditions, including our rodent studies of neonatal high oxygen (O_2_) exposure, show altered myocardial tissue with cardiomyocyte hypertrophy and fibrosis (Bensley et al. 2010; Bertagnolli et al. 2014).

In recent years, stem cells emerged as a novel and exciting therapeutic avenue to counteract tissue damage caused by injury or disease. Because of their capacity for self‐renewal and multi‐potency, stem cells are key contributors to development, organ repair and regeneration throughout life. Among the different types of progenitor cells, endothelial progenitor cells (EPCs) are critical for prenatal and postnatal development of organ systems (Asahara et al. 2011). Endothelial colony‐forming cells (ECFCs), a subset of EPCs with high self‐renewal and de novo vessel formation capacity, have been successfully tested experimentally in the treatment or prevention of bronchopulmonary dysplasia (BPD), the most frequent major complication of very preterm birth, characterized by lung vascular growth disruption (Alphonse et al. 2014). Whether ECFC treatment is also beneficial to myocardial changes observed after neonatal hyperoxic conditions is unknown. Considering the later, the current study aimed to examine the impact of exogenous human cord blood ECFCs treatment on heart morphological changes in the experimental model of O_2_‐induced cardiomyopathy.

## Materials and Methods

All experimental procedures were approved by the Animal Health Care Committee of the University of Alberta and the Animal Ethics Committee of the Sainte‐Justine University Hospital (CHU Sainte‐Justine) Research Centre, and followed the guidelines of the Canadian Council on Animal Care and the National Institutes of Health (NIH) Guide for the Care and Use of Laboratory Animals.

### Human umbilical cord blood ECFC isolation

Human umbilical cord blood ECFC isolation, expansion, and quality control were realized as reported (Mead et al. 2008; Alphonse et al. 2014). In brief, ECFCs were cultured from Ficoll‐separated layer of blood mononuclear cells seeded on rat tail collagen I precoated plates. After 4–7 days in culture, typical ECFCs well circumcised cobblestone‐appearing cell colonies were observed. Colonies were then isolated and expanded. ECFC characteristics were verified by fluorescence‐activated cell sorting to determine that early passage (Anversa et al. 1992; Asahara et al. 2011; Alphonse et al. 2014) cells expressed endothelial cell‐specific cell surface antigens CD31, CD105, CD144, and CD146, and were negative for hematopoietic cell‐specific antigen CD45 or the monocyte‐macrophage marker CD14 (all antibodies Abcam, Cambridge, MA). Phenotypic characterization of the cells also comprised AcLDL ingestion and their capacity to form capillary‐like structure in matrigel.

### Animal model and experimental procedures

Newborn Sprague–Dawley rat pups were exposed to high O_2_ concentration (95% O_2_, *n* = 15) from P4 to P14 using an oxycycler (ProOx P110, Biosherix, Lacona, NY). Dams were switched every 48 h with a surrogate mother of a litter maintained in RA, in order to avoid maternal morbidity associated with O_2_ toxicity and provide equal nutrition to each litter (Bertagnolli et al. 2014, 2016; Vadivel et al. 2014). We have previously shown that mother interchanges do not affect pup growth or survival (Yzydorczyk et al. 2008). Newborns from the control group were kept in RA, without mother interchange (21% O_2_, *n* = 13). Only males were studied, with a maximum of two offspring per condition per litter. At P14, eight pups from the O_2_‐exposed groups were injected, through the jugular vein, with human cord blood‐derived ECFCs (10^5^ cells/animal in 100 *μ*L of DMEM). Euthanasia was performed at P28 with intraperitoneal pentobarbital.

### Histology

Immediately after euthanasia, the hearts were removed, washed in potassium chloride (100 mmol/L KCl in saline) to induce diastolic arrest, and weighed. The hearts were fixed in 4% paraformaldehyde for paraffin embedding and histomorphometry analysis.

The transverse 5 *μ*m cross‐sections of the left ventricle (LV) and right ventricle (RV) were stained with hematoxylin and eosin to measure cardiomyocyte surface area or Masson's trichrome to evaluate cardiac fibrosis. For all histological analyses, three pictures per zone were obtained from three randomly chosen zones within the sub‐endocardium and the sub‐epicardium of the LV and of the RV, and the septum, for a total of 15 pictures per heart.

Cardiomyocyte size was evaluated by measuring the surface area of cells with a visible nucleus (*n* ≥ 20 cells/picture). Cardiac fibrosis and perivascular fibrosis (*n* ≥ 3 vessels/picture) were determined by quantifying the intensity of Masson's trichrome blue color staining (corrected as % of total pixels). Because the parameters measured did not differ between zones of the heart (sub‐endocardium, sub‐epicardium, septum for the LV; sub‐endocardium and sub‐epicardium for the RV), results from all zones (three images per zone) were averaged for each animal. Analyses were performed using Image J 1.36b (http://rsbweb.nih.gov/ij/) as described previously (Anversa et al. 1992; Der Sarkissian et al. 2003).

### Immunofluorescence

Samples were deparaffinized through three changes of xylene, and rehydrated in decreasing ethanol concentrations. After quenching in 0.1 mol/L glycin/PBS, antigen retrieval was first performed using citrate buffer solution (pH = 6.0), preheated at 65°C, and then by trypsin. Tissue sections were washed in 0.1% Triton X‐100/TBS, then blocked using BSA 3%/PBS. They were incubated overnight at 4°C with the primary antibody diluted in blocking solution, CD31 (M‐20, Santa Cruz Biotech; 1:100). The next day, they were treated for 1 h with the secondary antibody diluted in blocking solution: donkey anti‐goat IgG (DyLight 549, Abcam; 1:1000) for CD31. DAPI was used for nuclear DNA labeling. Immunostained slides were imaged by LED Inverted fluorescence microscope (DMIL LED Inverted Fluorescence Microscope, Leica, Germany) and images were analyzed using Image J 1.36b (http://rsbweb.nih.gov/ij/).

### Statistical analysis

Data are presented as mean ± SEM in the figures. Comparisons for O_2_ exposure and ECFC treatment were made using two‐way ANOVA followed by Bonferroni post‐hoc test. All statistical tests were carried out on the software GraphPad Prism version 7.04 for Windows (GraphPad Software, San Diego, CA). The significance level was set at *P < 0.05*.

## Results

### Morphometric parameters

Body weight did not significantly differ between groups throughout the study period. At P28 (age of sacrifice), heart weight and heart to body weight ratio also did not differ between groups (data not shown).

### Cardiac hypertrophy and fibrosis

**Figure 1:**
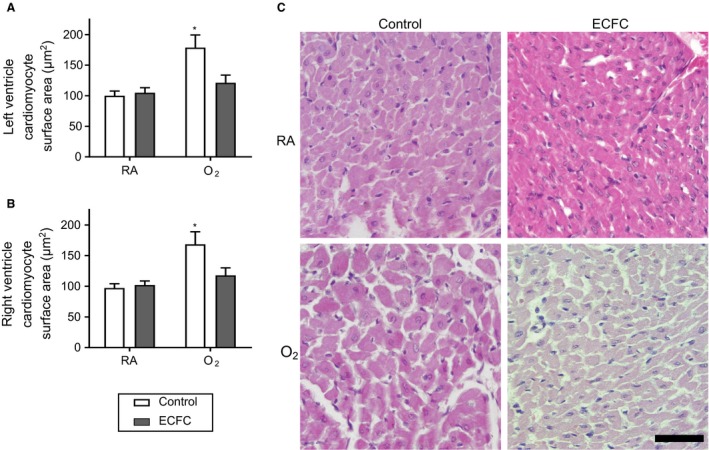
Effect of ECFC treatment on left and right ventricle cardiomyocyte hypertrophy. (A) Left ventricle and (B) right ventricle cardiomyocytes surface area (μm^2^) of 28 days old rats exposed to high concentration of oxygen (O_2_) versus room air (RA) in the neonatal period and subsequently treated with endothelial colony‐forming cells (ECFC) versus saline (Control). (C) Representative photomicrographs of LV cardiomyocytes stained with hematoxylin‐eosin. Magnification of X400; scale bar, 50 *μ*m. *P < 0.05 versus all other groups.

Cardiomyocytes surface area did not differ between the different zones of each ventricle (LV: sub‐epicardium, sub‐endocardium and septum; RV: epicardium and endocardium) so results were combined. O_2_‐exposed rats exhibited a significant increase in cardiomyocyte surface area in both the LV and RV (Fig.**** 1). ECFC treatment significantly attenuated the LV and RV cardiomyocyte hypertrophy in both ventricles of O_2_‐exposed animals, and was without effect in RA animals (Fig.**** 1).

**Figure 2:**
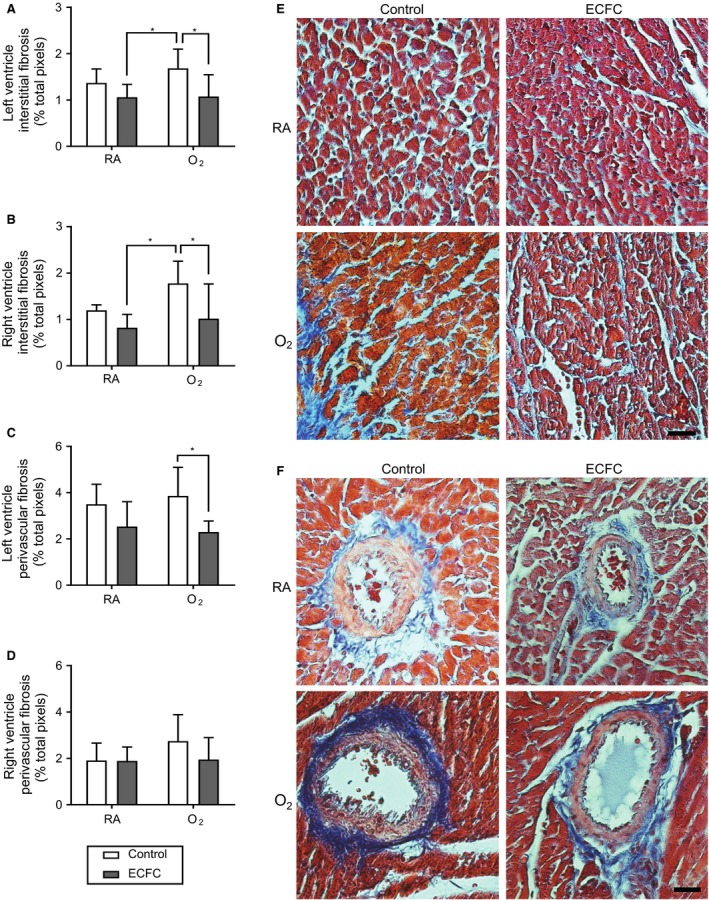
Effect of ECFC treatment on the left and right ventricle interstitial and perivascular fibrosis. (A) Left ventricle and (B) right ventricle interstitial tissue fibrosis (% total pixels) and (C) Left ventricle and (D) right ventricle perivascular fibrosis (% total pixels) of 28 days old rats exposed to high concentration of oxygen (O_2_) versus room air (RA) in the neonatal period and subsequently treated with endothelial colony‐forming cells (ECFC) versus saline (Control). (E) Representative photomicrographs of LV myocardium and (F) LV myocardium vessel stained with Masson's trichrome. Magnification of X400; scale bar, 50 *μ*m. *P < 0.05.

ECFC treatment was associated with a significant reduction in interstitial fibrosis (Masson's trichrome staining) in all LV and RV myocardium zones in O_2_‐exposed rats (Fig.**** 2). Specific assessment of perivascular fibrosis showed that ECFC treatment led to a significant reduction in the LV, but in not the RV (Fig.**** 2). ECFC treatment had no effect on tissue or perivascular fibrosis in RA rats.

### Cardiac marker of angiogenesis

**Figure 3:**
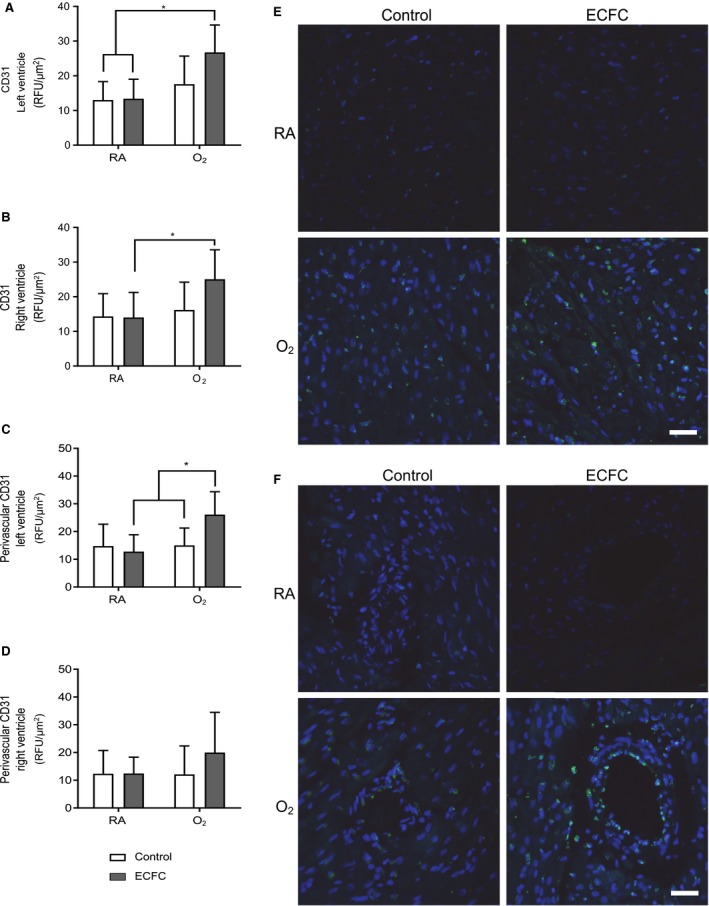
Effect of ECFC treatment on expression of angiogenesis marker CD31 (PECAM‐1) in left and right ventricle. (A) Immunofluorescence showing left ventricle and (B) right ventricle CD31 expression (RFU/μm^2^: relative fluorescence unit) and (C) Left ventricle and (D) right ventricle perivascular CD31 expression of 28 days old rats exposed to high concentration of oxygen (O_2_) versus room air (RA) and subsequently treated with endothelial colony‐forming cells (ECFC) versus saline (Control). (E) Representative photomicrographs of LV myocardium and (F) LV perivascular CD31 immunoflorescence. Magnification of X400; scale bar, 50 *μ*m. *P < 0.05; *P* = 0.08 O_2_‐ECFC versus O_2_‐Control in LV.

Myocardial tissue and perivascular CD31 expression did not differ between O_2_‐exposed and RA animals. However, ECFC treatment was associated with a significant increase in CD31 expression in LV and RV myocardial tissue and in LV perivascular (but not RV) of O_2_‐exposed animals; whereas it had no impact in the RA group (Fig.**** 3).

## Discussion

As previously reported, our current study found that cardiomyocyte surface area and interstitial fibrosis of both ventricles were increased in juvenile (28‐days old) rats exposed to high O_2_ as newborns (Bertagnolli et al. 2014, 2016). Perivascular fibrosis was also increased, which we had not assessed in our previous studies. Current results showed that iv injection of ECFCs at P14, at the end of hyperoxia exposure, effectively prevented cardiomyocyte hypertrophy and myocardial and perivascular fibrosis in the O_2_‐exposed group while having no effect in the RA group. ECFC treatment was also associated with an increased expression of CD31, a marker of angiogenesis, in LV and RV myocardial tissue and in LV perivascular area of O_2_‐exposed compared to O_2_‐exposed‐vehicle‐treated animals. RA animals injected with ECFCs did not display an increase in CD31.

Neonatal hyperoxia in rodents is a well‐recognized model of preterm birth‐related conditions, most commonly used to study complications such as BPD or retinopathy of prematurity (Sapieha et al. 2010; Buczynski et al. 2013; O'Reilly and Thebaud 2014; Winners‐Mendizabal et al. 2014). Expanding the study of this model, we have reported on long‐term cardiovascular impact after neonatal exposure to high O_2_ including increased blood pressure, cardiomyocyte hypertrophy, and increased cardiac fibrosis (Yzydorczyk et al. 2008; Bertagnolli et al. 2014, 2016). In the present study, we did not observe the slight increase in heart to body weight index in O_2_‐exposed animals reported previously (Bertagnolli et al. 2014, 2016). However, comparable to current findings, a recent study using a similar neonatal hyperoxia model showed no difference in cardiac hypertrophy index at 1 year (Goss et al. 2017), indicating that the heart to body weight index might not be sensitive to fine changes in cardiac cell structure and function observed in this model.

The therapeutic use of stem or progenitor cells is a rapidly expanding field of study in prematurity‐related conditions. Cell‐based therapies, using different types of progenitor cells including ECFC, early outgrowth endothelial progenitor cells, mesenchymal stem cells (MSC), and vascular progenitor cells have been investigated in animal models of BPD and showed promising results to counteract the effect of hyperoxia or inflammation‐induced neonatal lung injury (O'Reilly and Thebaud 2012; Borghesi et al. 2013; Bohlin 2016). MSC and MSC‐conditioned media as a cell therapy for BPD were found to prevent lung vascular and alveolar changes observed in hyperoxia‐induced lung injury in mice and rats (Aslam et al. 2009; van Haaften et al. 2009; Hansmann et al. 2012; Pierro et al. 2013). More recently, Alphonse et al. (2014) showed that ECFC and ECFC‐derived conditioned media reduced lung parenchymal and vascular damage caused by neonatal O_2_ exposure in rodents. However, the impact of cell therapy on myocardial changes in experimental models of prematurity‐related conditions has not been, to our knowledge, reported. Indirect evidence indicates that RV changes could be prevented or reversed by cell therapy. ECFC therapy in O_2_‐exposed immunocompromised rag^−/−^ mice reduced pulmonary hypertension and decreased RV hypertrophy in addition to improving lung compliance and alveolar architecture (Alphonse et al. 2014). In RNU nude rats (another BPD model), ECFC reinstated alveolar architecture and vascular growth in the lungs, and decreased RV hypertrophy (Alphonse et al. 2014).

Our study thus provides the first evidence that administration of exogenous‐derived ECFC attenuates cardiac morphological changes following neonatal hyperoxia. While our observed improvement on fibrosis and cardiomyocytes hypertrophy in the RV following ECFC treatment might be extrapolated to be caused by a decrease in pulmonary vascular resistance, the same cannot be presumed about the LV. Indeed, the increase in systemic blood pressure in hyperoxia‐exposed rats was not detectable at 28 days, making it unlikely for an improvement in blood pressure to account for LV improvement in the ECFC‐treated rats (Yzydorczyk et al. 2008; Bertagnolli et al. 2014, 2016). Beyond the lungs, neonatal hyperoxia affects vascular development in the retina and in peripheral striated muscles (Yzydorczyk et al. 2008; Sapieha et al. 2010). In experimental neonatal models, human circulating EPCs were shown to normalize vascular growth in retinopathy of prematurity and cord blood ECFC improved long‐term neurological exam in hypoxic ischemic encephalopathy (Wang et al. 2016; Grandvuillemin et al. 2017). Whether ECFC treatment also ameliorates impaired systemic microvascular development, and perhaps peripheral resistance, remains to be studied.

Improved cardiomyocytes hypertrophy that we observed after ECFC therapy may also be related to promotion of coronary angiogenesis. Indeed, CD31 immunostaining, a marker of angiogenesis, is increased in both ventricles myocardial tissue and in the LV perivascular area of ECFC‐treated O_2_‐exposed animals in comparison with control groups. Angiogenesis is necessary for reversal of pathological cardiac hypertrophy (Hou and Kang 2012). ECFC and ECFC‐derived exosomes administration in experimental models of cardiac diseases decreases hypertrophy, diastolic dysfunction, and fibrosis through improved angiogenesis and reduced expression of profibrosis genes (Eirin et al. 2015; Kim et al. 2016; Ke et al. 2017).

Interestingly, RA control animals treated with ECFCs did not display an augmentation of CD31 marker. The later observations are consistent with the literature showing higher expression of this marker following ECFC treatment localized specifically in regions impaired by the experimental protocol, i.e., regions to be rescued by the treatment. In an ischemic stroke mouse model, 15 days after ECFC injection, expression of angiogenesis marker CD31 was significantly higher in the ischemic border region when compared to the same region in vehicle‐treated animals (Ding et al. 2016). Whether this observation results from a selective response to an injury signal remains to be determined.

Clinical trials addressing heart failure or myocardial ischemia have used, for most, unfractionated autologous bone marrow‐derived mononuclear cell transplantation and were not successful at demonstrating clinically significant improvement (Nguyen et al. 2016). Circulating ECFC increase significantly after cardiac injury (Massa et al. 2005). Whereas no clinical trial has directly examined ECFC treatment in cardiac disease, the proportion of ECFC in autologous bone marrow‐derived mononuclear cell transplantation was shown to be lower in a subgroup analysis of patients with the most pronounced cardiac improvement after therapy. This finding was interpreted as evidence for enhanced ECFC mobilization from the bone marrow toward to injured myocardium (Taylor et al. 2016).

Current results also show that ECFC treatment decreased overall myocardial fibrosis as well as perivascular fibrosis in the LV. Perivascular fibrosis of the aorta was also reported in this model (Huyard et al. 2014). Myocardial fibrosis, and particularly perivascular fibrosis, is increasingly recognized a key driver in the development of cardiac dysfunction, and there are few effective antifibrosis therapies (Ytrehus et al. 2018). Interestingly, while treatment with both human umbilical cord blood MSC and ECFC improved function and vascularization of infarcted myocardium in rats, only ECFC improved tissue fibrosis (Kim et al. 2016). In studies of adult cardiac diseases, it remains unclear whether perivascular fibrosis is cause or consequence of hypertension‐ or ischemia‐associated cardiac remodeling and dysfunction (Ytrehus et al. 2018). Taken together, current and previous studies suggest that fibrosis occurs concomitantly with cell hypertrophy in cardiomyopathy associated with neonatal hyperoxia exposure and might be triggered by common pathway, prior to the development of hypertension (Yzydorczyk et al. 2008; Bertagnolli et al. 2014, 2016).

The mechanistic pathways specifically underlying the beneficial effects of ECFC that we observed in the developing myocardium can comprise local tissue and/or perivascular engraftments as well as paracrine effects, including through the release of exosomes or microparticles, but these were not explored in current studies (Schwarz et al. 2012; Burger et al. 2015; Kim et al. 2016).

In summary, the current study unveils new insights into the therapeutic potential of ECFC for the management of long‐term cardiovascular consequences of preterm birth. Exogenous administration of ECFCs was shown to attenuate cardiac morphological changes induced by neonatal hyperoxic stress, possibly in part through promotion of angiogenesis in the heart. In light of these findings, short‐ and long‐term effects of ECFC‐based therapy on cardiac function and other cardiovascular outcomes (including peripheral vascular changes and elevated systemic blood pressure in adulthood) need to be assessed, and mechanisms underlying ECFC activity after deleterious neonatal conditions, more specifically in the heart, require further investigation.

## Conflict of Interest

None.
